# African swine fever outbreak on a medium-sized farm in Uganda: biosecurity breaches and within-farm virus contamination

**DOI:** 10.1007/s11250-016-1197-0

**Published:** 2016-12-13

**Authors:** Erika Chenais, Susanna Sternberg-Lewerin, Sofia Boqvist, Lihong Liu, Neil LeBlanc, Tonny Aliro, Charles Masembe, Karl Ståhl

**Affiliations:** 1 National Veterinary Institute, Uppsala, Sweden; 2 Swedish University of Agricultural Sciences, Uppsala, Sweden; 3 Directorate of Production and Marketing, Gulu District Local Government, Gulu, Uganda; 4 Makerere University, Kampala, Uganda

**Keywords:** Farm biosecurity, Environmental sampling, Environmental contamination, Infectious disease outbreak, Smallholders

## Abstract

In Uganda, a low-income country in east Africa, African swine fever (ASF) is endemic with yearly outbreaks. In the prevailing smallholder subsistence farming systems, farm biosecurity is largely non-existent. Outbreaks of ASF, particularly in smallholder farms, often go unreported, creating significant epidemiological knowledge gaps. The continuous circulation of ASF in smallholder settings also creates biosecurity challenges for larger farms. In this study, an on-going outbreak of ASF in an endemic area was investigated on farm level, including analyses of on-farm environmental virus contamination. The study was carried out on a medium-sized pig farm with 35 adult pigs and 103 piglets or growers at the onset of the outbreak. Within 3 months, all pigs had died or were slaughtered. The study included interviews with farm representatives as well as biological and environmental sampling. ASF was confirmed by the presence of ASF virus (ASFV) genomic material in biological (blood, serum) and environmental (soil, water, feed, manure) samples by real-time PCR. The ASFV-positive biological samples confirmed the clinical assessment and were consistent with known virus characteristics. Most environmental samples were found to be positive. Assessment of farm biosecurity, interviews, and the results from the biological and environmental samples revealed that breaches and non-compliance with biosecurity protocols most likely led to the introduction and within-farm spread of the virus. The information derived from this study provides valuable insight regarding the implementation of biosecurity measures, particularly in endemic areas.

## Introduction

African swine fever (ASF) was first observed and described in pigs of European settlers (*Sus scrofa*) in Kenya in the beginning of the twentieth century (Montgomery 1921). Close to a century later, the disease is present in most pig-keeping areas on the African continent (Penrith and Vosloo 2009). ASF is a contagious, typically lethal, hemorrhagic disease of domestic pigs caused by a double-stranded DNA virus, the sole member within the *Asfarviridae* family, genus *Asfivirus* (Plowright et al. 1994). A common clinical presentation in sub-Saharan Africa is peracute or acute hemorrhagic fever with almost 100% case fatality rate (Plowright et al. 1994). The epidemiology is complex with a sylvatic cycle involving asymptomatically infected warthogs and soft ticks, a domestic cycle involving soft ticks and domestic pigs, and an additional domestic cycle with pig-to-pig transmission (Costard et al. 2013; Jori et al. 2013). The latter transmission cycle has been identified as the main driver of disease in areas with a high density of pigs, mainly free-range systems, and a low level of farm biosecurity such as in sub-Saharan Africa (Penrith et al. 2013). ASF has severe economic impacts, both in high- and low-income countries (Sanchez-Vizcaino et al. 2013; Mur et al. 2014; Chenais et al. 2015a), and its control is essential for profitable pig production. Achieving control is complicated as ASF virus (ASFV) may remain viable for long periods in infected pig tissues, meat, and processed pig products, and by the lack of an effective vaccine. However, it has been shown that control can be realized through strict compartmentalization separating domestic pigs from the sylvatic hosts and by targeted biosecurity (Penrith and Vosloo 2009).

In Uganda, a low-income country in sub-Saharan Africa, ASF is endemic. The presence of ASFV in the sylvatic cycle is probably as ancient as in other areas of the region (Montgomery 1921; Plowright et al. 1994), but with a growing domestic pig population, numerous outbreaks are now described every year (Gallardo et al. 2011; Muwonge et al. 2012; Atuhaire et al. 2013; Barongo et al. 2015; Chenais et al. 2015b; Muhangi et al. 2015). The Ugandan pig population is the largest in East Africa (FAOSTAT 2013), but larger-scale enterprises are rare and most of the pigs are still kept in smallholder family farms in the rural areas (NEPAD and FAO 2004; Dione et al. 2014).

In the smallholder subsistence farming systems that dominate in low-income countries such as Uganda, farm biosecurity is practically non-existent (Costard et al. 2009a; Fasina et al. 2012; Dione et al. 2015; Leslie et al. 2015; Nantima et al. 2016). The majority of pigs roam freely at least parts of the year, and even if an enclosure for the pigs is available, animals are frequently found outside the pens (Dione et al. 2014; Ikwap et al. 2014; Chenais et al. 2015a). As the pigs are not confined, restriction of visitor’s access to the animals, change of clothing and boots, insect and rodent control, quarantine of new animals, environmental, feed, and water hygiene or any other biosecurity measures become difficult to achieve (Young et al. 2013; Dione et al. 2015). Poverty prevents even simple investments and promotes utilization of all animal protein. The consequences are consumption of meat from animals that have died or been slaughtered upon showing signs of disease, and trade in sick animals or animals that have been in contact with sick animals (Chenais et al. 2015a; Leslie et al. 2015; Nantima et al. 2016). The lack of biosecurity and related continuous circulation of ASF in smallholder settings in endemic areas thus creates a biosecurity challenge for enterprises that aspire at larger-scale pig farming in these areas. In smallholder settings in endemic areas, outbreaks of ASF, like other diseases, are largely under-reported (de Balogh et al. 2013; Chenais et al. 2015a; Nantima et al. 2015). As a consequence, the progression of outbreaks and the related impacts are seldom described in detail. In order to attempt control of ASF, epidemiological knowledge is needed on the global, national, regional, and local scale, including individual outbreaks, i.e., on farm level (FAO 2014). If larger-scale pig farming in ASF-endemic areas in resource-poor settings such as sub-Saharan Africa are to succeed, these knowledge gaps need to be filled and the associated specific challenges better understood.

The objective of this study was to investigate and describe an outbreak of ASF on farm level in an endemic area, including investigations of the on-farm environmental virus contamination during the outbreak. Based on this, we discuss challenges facing pig farming in endemic areas, with special emphasis on farm-level biosecurity.

## Materials and methods

The study was carried out on a pig farm in Lira district in northern Uganda in March, April, and September 2014.

### Study area

Lira district, in northern Uganda, has a human population of about 400,000 and covers approximately 3500 km^2^ of land (UBOS 2014). Since the civil conflict between 1987 and 2007, the human population is heavily concentrated in Lira town (Jacobsen et al. 2006). The most recent livestock census dates from 2008, and at that time, the district held 28,630 pigs (UBOS 2008). Since then, all indications are that the number of pigs has increased.

### Study farm

The study farm was run by a non-governmental organization (NGO) and established to financially support the organization’s humanitarian activities. The objective of the farm was to produce piglets and pork and in addition to offer training in pig husbandry for local pig farmers. As such, the farm represented a larger-scale farming operation located in an area with typical smallholder family farms, thus vulnerable to the various challenges, including biosecurity, facing a pig farm in an ASF-endemic setting.

**Fig. 1:**
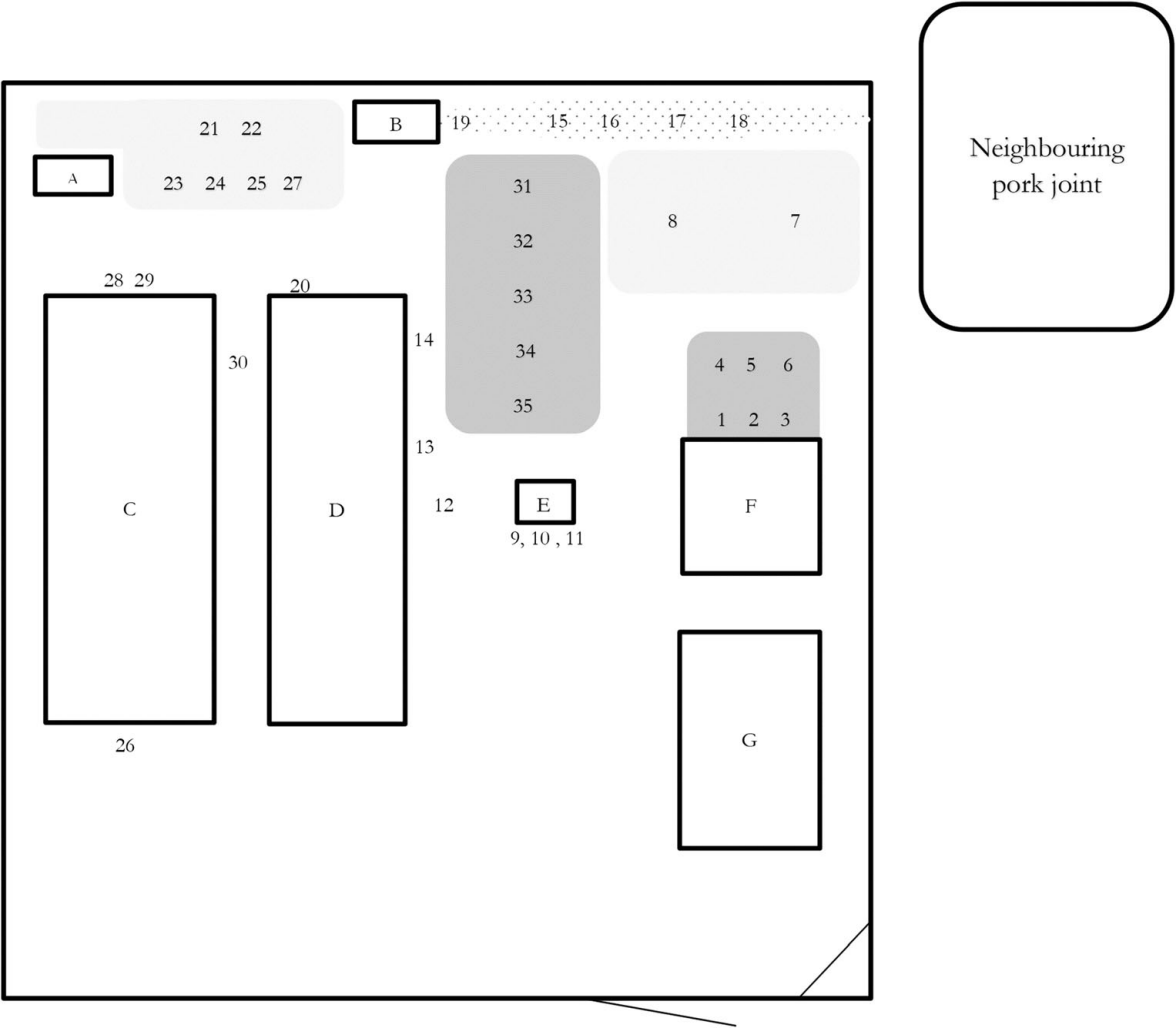
Approximate plan of the study farm indicating sites where environmental samples were taken during a confirmed African swine fever outbreak in a medium-sized farm in Lira district, Uganda. *Lettered squares* mark the different buildings, *light gray* areas indicate sites used for placing carcasses awaiting burial, and burial of carcasses, and *dark gray* areas mark sites used for bleeding pigs at slaughter. The *dotted* area outlines the overflow from a septic tank. *Numbers* mark environmental samples (1–35) taken on the 2nd of April 2014. Compound size: around 40 × 60 m. *A* latrines; *B* septic tanks; *C* concrete pig stables, 10 pens; *D* wood fence pig stables, 3 pens; *E* first slaughter place, wood structure; *F* latter slaughter place, concrete slab; *G* offices and storage

The farm started breeding pigs in 2013. In January 2014, the farm had 35 adult pigs and 103 piglets and growers, all of exotic (typical European white landrace) breed. The pigs were kept in a purpose-built, fenced compound with two stables. One stable was made entirely of concrete with a tin roof (“concrete stable”), and one had concrete floors, tinned roof, and pen walls of wooden planks with gaps between the planks allowing nose-to-nose contact (“wood fence stable”). The compound also contained additional buildings for office, storage, and guards and an unfinished construction with a cement slab (see Fig. 1). After slaughter, the meat was transported to the office of the NGO for storage, sale, or consumption. At the end of January 2014, the farm started selling and slaughtering the first animals. In the first week of March 2014, the authors were contacted concerning disease and mortalities in the pigs. Already at this stage, ASF was suggested based on the clinical signs and the location in northern Uganda, and some preliminary advice to contain the disease and prevent further spread was given.

### Data and sample collection

The study included five farm visits (11th of March, 23rd of March, 2nd of April, 10th of April, and 21st of September 2014) comprising interviews with farm representatives, site assessment, and the collection of biological and environmental samples. Interviews were informal, with simultaneous note-taking. Photographs were taken as part of the assessment, and feedback in the form of investigation reports were provided to farm representatives following each visit. Information regarding biosecurity was extracted from the interview data and from observations made during the farm visits.

Biological samples were collected on the 11th of March and 10th of April 2014 (see Table 1). Blood and serum were obtained by puncture of the jugular vein using a vacutainer system. Organ samples were obtained from the carcass of one animal. Five to ten grams of material was collected from the liver, kidney, spleen, and lung, respectively, and put in separate, disposable plastic containers.

**Table 1:**
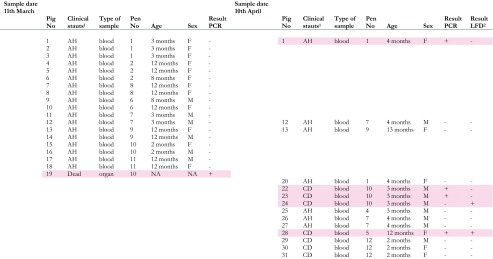
Biological samples taken on the 11th March and 10th April 2014 on a medium-sized farm in Lira district, Uganda, and analyzed for the presence of African swine fever virus nuclear acids (samples from the 11th of March) or African swine fever virus nuclear acids and ASF antibodies (samples from the 10th April)

Samples with positive results for either test are marked in pink

*NA* data not available, *AH* apparently healthy, *CD* clinically diseased, *LFD* lateral flow device

Environmental samples were taken on the 2nd of April 2014. Places to be sampled were chosen based on visible blood contamination and from interviews indicating biosecurity high-risk spots. Sample material was in most cases soil with some samples of feed, water, manure, or pig hair (Table 2). Each sample consisted of approximately 15–30 cl of material. One pair of disposable gloves and one disposable spoon were used for each sample. Samples were put in individual disposable plastic containers.

**Table 2:** Environmental samples taken on the 2nd of April 2014 on a medium-sized farm in Lira district, Uganda, and analyzed for the presence of African swine fever virus nuclear acids

Sample number	Material	Location/description	Result PCR
1–3	Soil	In front of the concrete construction used for slaughter	+
4–6	Soil	Site where blood have been buried	+
7–8	Soil	Burial ground	+
9	Hair/other remains	On wooden structure previously used as slaughter slab	+
10–11	Soil and hair	Under wooden structure previously used as slaughter slab, partly burnt	+
12	Soil	Original placement of wooden structure previously used as slaughter slab	+
13	Soil	On the ground outside pen no. 12, cleaning water overflow at this point	+
14	Soil	On the ground outside pen no. 13, cleaning water overflow at this point	+
15–19	Soil/manure	Overflow from septic tank	+
20	Water/feed	Cleaning water from outdoor pens	+
21–24	Soil	Burial ground between latrines/fence/septic tank	+
25	Soil	Burial ground between latrines/fence/septic tank	−
26	Soil	Outside cement pig stable, next to disinfection foot bath, “entrance”	+
27	Soil	Under a dead pig on burial ground	−
28–29	Soil	Outside cement pig stable, next to disinfection foot bath, “exit”	+
30	Soil	Between concrete and outdoor pens	+
31	Soil	Location where pigs have been bled at slaughter	+
32	Soil	Location where pigs have been bled at slaughter	+
33	Soil	Location where pigs have been bled at slaughter	+
34	Soil	Location where pigs have been bled at slaughter	+
35	Soil	Location where pigs have been bled at slaughter	+

Biological samples were stored overnight in a fridge at the district veterinary office in the neighboring Gulu district before transport. Environmental samples were transported directly to the laboratory at Makerere University in Kampala. During transport, samples were kept cool with ice in a cooler bag. On arrival to the laboratory, serum samples were centrifuged and sera separated. Serum, blood, and environmental samples were stored at −20 °C until further processing.

### Laboratory investigations

Laboratory analyses were done at the Molecular Biology Laboratory at Makerere University, in Kampala, and the national reference laboratory, National Animal Disease Diagnostics and Epidemiology Centre in Entebbe. DNA extraction of biological samples was done either by DNeasy Blood and Tissue kit (QIAGEN AG, Hombrechtikon, Switzerland) (samples from the 11th of March) or by a MagMAX kit (Thermo Fisher Scientific Inc., Waltham, MA, USA) (samples from the 10th of April), according to the instructions of the manufacturers. All organ material was pooled before DNA extraction. The extractions from the 11th of March were analyzed for the presence of ASFV nucleic acids using a commercially available real-time PCR assay (Tetracore ASFV; Tetracore Inc., Rockville, MD, USA) according to the instructions of the manufacturer. The extractions from the 10th of April were analyzed for the presence of ASFV nucleic acids with a Universal Probe Library (UPL) probe (5′-FAM-GGCCAGGA-dark quencher-3′) (Roche Diagnostics, Rotkreuz, Switzerland) as previously described (Fernandez-Pinero et al. 2013). DNA from the environmental samples was extracted using the PowerLyzer® PowerSoil® DNA Isolation Kit (MO BIO Laboratories Inc., Carlsbad, CA, USA) in accordance with the instructions of the manufacturer. The DNA extractions of the environmental samples were analyzed for the presence of ASFV nucleic acids using a commercially available real-time PCR assay with internal control (IC) (Tetracore ASFV; Tetracore Inc., Rockville, MD, USA) in accordance with the instructions of the manufacturer.

Blood samples taken on the 10th of April were also tested for the presence of ASF antibodies using a lateral-flow device test (Ingenasa, Madrid, Spain) in accordance with instructions of the manufacturer.

## Results

### Outbreak

**Fig. 2:**
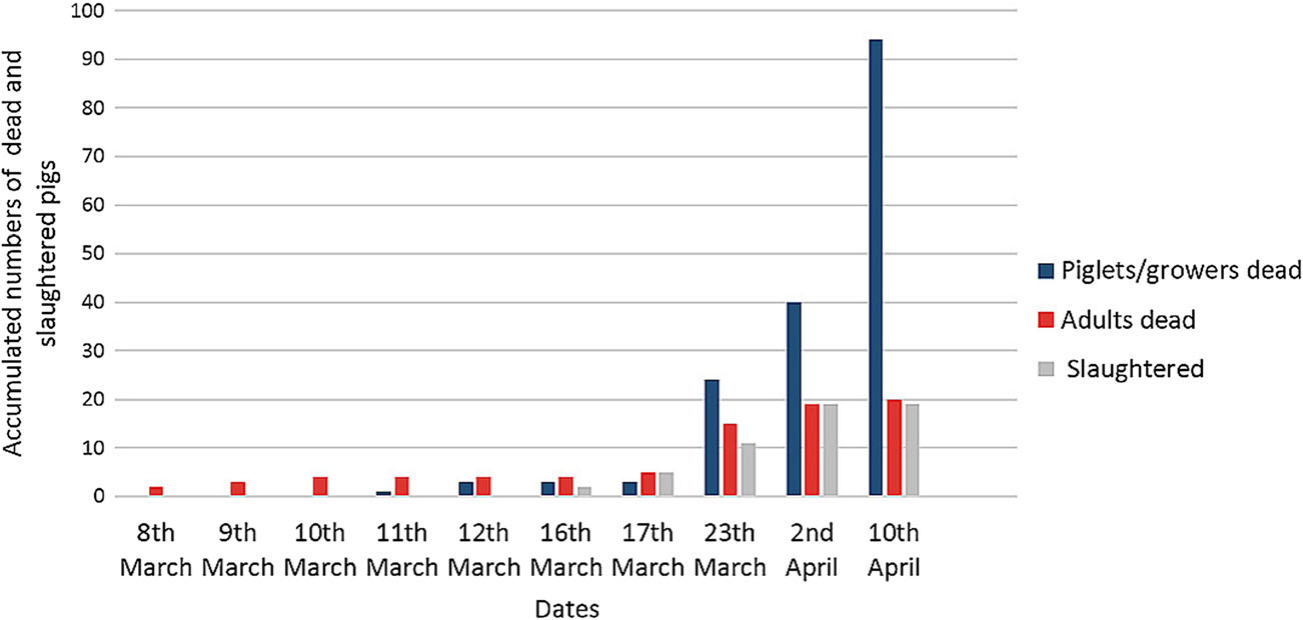
Accumulated number of dead and slaughtered pigs during a confirmed African swine fever outbreak in a medium-sized farm in Lira district, Uganda

The outbreak became apparent on the 7th of March 2014 with an adult boar presenting with shivering, fever (40.8 °C), and anorexia (Table 3). The boar had escaped from its pen into the yard 6 days earlier. The next day, two sows aborted, and one of them plus the aforementioned boar died. The other sow died the day after. The following days, a few pigs died each day, most of them adult pigs. Between days 10 and 16 of the outbreak, many piglets/growers died, and the cumulative death figures started to show signs of exponential growth (see Fig. 2). On the 34th day of the outbreak, 133 pigs had either died or been slaughtered upon showing clinical signs of ASF. Three months after the onset of the outbreak, all pigs at the farm had either died or been slaughtered and the farm was empty.

**Table 3:** Temporal description of a confirmed African swine fever outbreak in a medium-sized farm in Lira district, Uganda

Date	Day of outbreak	Event	Accumulated number of dead and slaughtered pigs
7th March	0	First pig shows clinical signs: fever, shivering, anorexia	0
8th March	1	First pig dies, 2 sows abort, 1 of these dies	2
9th March	2	Second sow that aborted the day before dies	3
10th March	3	Fourth adult pig dies	4
11th March	4	First piglet/grower dies	5
12th March	5	2 growers die	7
16th March	9	2 adult pig show clinical signs, both slaughtered	9
17th March	10	1 adult pig dies, 3 adult pigs start showing clinical signs, all 3 slaughtered	13
23rd March	16	In total 15 adult pigs have died, 11 adult pigs have been slaughtered, and 24 piglets/growers have died	50
2nd April	26	Since the 23rd of March, 4 adult pigs have died, 8 adult pigs have been slaughtered, and 16 piglets/growers have died	78
10th April	34	Since the 2nd of April, 54 piglets/growers and 1 adult pig have died	133
June		All pigs dead or slaughtered	>138

### Laboratory investigations

A DNA extraction of the pooled organ samples from one dead pig from the 11th of March was positive for the presence of ASFV. All 18 DNA extractions of blood samples from the 11th of March were negative for the presence of ASFV. Five DNA extractions of blood samples from the 10th of April were positive for ASFV. One of these ASFV-positive samples was also positive for the presence of ASF antibodies, as was one of the ASFV-negative samples. See Table 1 for details of the results of analyses of biological samples and the clinical status of the sampled pigs. Out of 35 DNA extractions of the environmental samples, 33 were positive for the presence of ASFV (Table 2).

### Biosecurity challenges

The first impression of the farm setup was that the farm seemed to comply with rather high biosecurity standards and to be fit for pig farming in an ASF-endemic area. The fenced and guarded compound, staff wearing overalls and rubber boots, and pigs confined in pens and fed only concentrate feed contributed to this impression. However, the interviews and more in-depth assessment of the farm site revealed that biosecurity standards were lacking, including routines as well as the layout and location of specific activities. Specific biosecurity risks are described in more detail below.

#### Slaughter

The slaughter took place inside the compound, less than 3 m from the wood fence stable, with no drainage and on an unpaved surface (see Fig. 1). During the outbreak, adult pigs and growers were slaughtered upon showing signs of disease in order not to lose the entire value of the pig. At slaughter, pigs were stunned with a gun and the throat was cut when the animal was lying on the ground. Excess blood was directed towards an excavated hole at the slaughter site and buried together with the offal. After slaughter, the carcasses were placed on a wooden structure during evisceration, cleaning, and butchering. Water was used to wash the carcasses, but there was no system for collecting this water. After the visit on the 23rd of March, slaughter was moved to the concrete slab (see Fig. 1). At the visit on the 23rd of March and 2nd of April, pieces of meat and pig hair could be found on the ground around the slaughter sites, demonstrating a lack of appropriate hygiene standards. The slaughter was performed by the same personnel that tended to the pigs. After the beginning of the outbreak, overalls were changed and boots were cleaned and put in the disinfection boot bath between slaughter and tending to live pigs.

#### Personnel

At the visit on the 11th of March, four different staff members tended to the pigs, including the guards. There had been a high turnover of staff with four farm workers leaving in the previous 6 months, causing biosecurity routines to be lost in the transition process. During the course of the outbreak, the manager quit his position, leaving only less experienced personnel behind. According to the routines, rubber boots and overalls were provided for the staff and overalls were washed daily. Despite the stated routines, it was apparent that procedures were not being followed, as some personnel were not using overalls at the visit on the 23rd of March.

#### Water management

Pig pens were cleaned with a water hose and the cleaning water tunneled towards the manure tank. Part of the water from cleaning of the wood-fence stable was flowing on the ground along the side of the stables. The manure tank was overflowing, creating a manure-filled ditch close to the fence at one side of the compound (see Fig. 1).

#### Pig records and movements

Pigs were not individually marked, and prior to the outbreak, no records were kept. Pigs that fell sick during the outbreak were in general not euthanized or slaughtered immediately but left in the pen, or moved to pens with other sick pigs. Dead pigs were buried within the compound, and while awaiting burial, they were left on the ground for several hours. During cleaning of the pens, pigs sometimes escaped into the yard where they had access to the slaughter site and the overflow from the manure tank.

#### Stable hygiene

When the outbreak started, disinfectant boot baths were placed at both entrances to the concrete stable and at the gate to the compound. After the visit on the 11th of March, a commercial disinfectant with ammonium chloride as the active ingredient was used. However, the boot baths were placed on an unpaved surface and used without prior cleaning of the boots, causing contamination of the water by organic material. In both sites, the boot baths were placed without any demarcation of the “clean” and the “dirty” side. General cleaning of the pens seemed to be insufficient as material from an abortion was found in a pen at the visit on the 23rd of March.

#### Other

Offal from slaughter and dead pigs was buried in a rather limited area of the compound. As the number of deaths and the related slaughter of sick pigs increased, the risk from these burials (digging in already used plots and the resurfacing of still active ASFV) increased. In January 2014, 2 months prior to the outbreak, the closest neighbors of the farm, just adjacent to the fence, opened a pork joint (in Uganda—a restaurant specialized in roasting pork) with on-site slaughter. Offal from slaughter at the pork joint was left in the open.

### Social and economic impacts and challenges

Before the outbreak, the farm employed two farm workers, two guards, and a farm manager. At the visits on the 23rd of March and the 2nd of April, the farm manager expressed feelings of deep stress and depression from seeing the pigs getting sick and die and not being able to control the outbreak. At the visit on the 10th of April, the farm manager had left his position without giving notice to the employer. On the visit of the 21st of September, the farm representative reported that he had considered leaving his position because of similar feelings of stress and depression connected to the outbreak. Apart from the farm manager who left his post voluntarily, two staff members lost their job due to the outbreak as no farm labor was needed once all pigs had died. The staff at the NGO’s main office also expressed worries that they might lose their employment due to the financial impacts of the outbreak.

In total, 35 adult pigs and more than a hundred piglets or growers died or were slaughtered with clinical symptoms of ASF during the outbreak. The farm estimated the market value of a pig at slaughter to 90 USD. The full value of all pigs were not lost as some were slaughtered. Further, the planned income was reduced by more than the slaughter value of the herd pre-outbreak, as a fourth of the original herd was breeding stock not meant for slaughter and representing a higher value. The farm estimated the total loss (including costs for re-stocking, de-contamination, repair, and extra work, but excluding the gain made from slaughter during the outbreak) at approximately 20,000 USD.

## Discussion

The results from the outbreak investigations correspond to the pathogenesis and survival features of ASFV. The virus can be found in a wide range of tissues and secretions, including feces and urine, from clinically affected pigs (Greig and Plowright 1970; Plowright et al. 1994; Davies et al. 2015). The highest viral loads are found in blood (Blome et al. 2013). Virus can be isolated from blood and organs before the pigs show any clinical signs (Gallardo et al. 2015). Feces has been shown to remain infectious for 1 week, even longer if kept dark (Montgomery 1921; Davies et al. 2015). However, ASFV is quickly inactivated in slurry (Turner et al. 1999). In African conditions, virus that is unprotected by organic material is inactivated within a couple of days if exposed to sunlight (Montgomery 1921). Based on the known physiology of the virus, following the initial introduction onto the farm, several routines observed in this study constituted a high risk for sustaining the outbreak within the farm. The presence of ASFV nucleic acids at various sites and in various environmental specimens (soil, water, feed, pig hair, manure) demonstrates the high level of contamination that occurred and the failure of the biosecurity routines that were employed.

The source of ASF introduction was never determined. One possible source could be infected offal or pork from the neighboring pork joint, brought into the farm by birds, rodents, or humans. The fact that the primary case had escaped into the yard some days before disease onset indicates the presence of virus in the yard. The high staff turnover, combined with a lack of external biosecurity, were other risk factors for introduction of ASF. Staff may have had pigs of their own at home, or in their villages, allowing for indirect virus transmission via, e.g., shoes. Prior to the outbreak, boot baths were not used. Rubber boots were provided for the farm workers, but as no biosecurity barrier with change of boots protocol was in place, ASFV could easily have been brought in by any farm worker, vehicle, or visitor. Boot baths that were put in place after the start of the outbreak were most probably ineffective due to contamination with organic material (Amass et al. 2000). In order for boot baths to be effective, boots need to be cleaned from organic material prior to disinfection and to rest in the boot bath between 2 and 5 min (depending on disinfectant used) (Amass et al. 2000; Amass and Ragland 2001). A change of boots is a safer, quicker, more environmentally friendly, and also cheaper (the cost of a pair of rubber boots in Uganda is approximately 5 USD) alternative than boot baths.

The process of slaughtering inside the compound constitutes a very high biosecurity risk. In this case, the risk pertained not only to slaughter in immediate proximity to the pigs but also to the fact that slaughter was performed on a soft surface without drainage and with no way to safely dispose of offal. Moreover, the same staff took care of the live pigs and performed the slaughter. This represents a biosecurity failure in both infrastructure and protocols. A safe slaughter process in general, and even more importantly when slaughter takes place in close connection with pig farming, requires a strict one-way flow from the dirty (live pigs) to the clean (meat) side (Skaarup 1985). No equipment, animals, or personnel must move from the dirty to the clean side, and no movement back to live pigs from neither clean nor dirty side can be allowed. Facilities and equipment that are easily cleaned, proper drainage, and safe disposal of offal, blood, and water from the slaughter process are essential for managing the risk of transmission of many pathogens (Skaarup 1985). Performing safe slaughter within a pig farming compound in an ASF-endemic area is very challenging and requires extremely strict biosecurity routines in order to reduce the biosecurity risks to acceptable levels.

In the described outbreak, most pigs had died within a month and all pigs had died or been slaughtered within three months. Farm records did not allow full distinction between pigs that died by themselves from the disease, those slaughtered upon showing clinical signs, or those slaughtered as a preventive action to avoid losses. The time to clearance of the outbreak (meaning no infectious animals or virus are left on the farm), however, matched recent simulations on the spread of ASF on farm level for pig herds of the same size, based on the ASFV strain from Georgia (Halasa et al. 2016). In the model developed by Halasa et al. (2016), time to clearance is significantly correlated to herd size and, depending on variables, related to the transmission rate per day as well as the infectiousness of residues of dead animals and of subclinically infected animals. The infectiousness of material and infected animals will not be different in this setting compared to other outbreaks, but the breaches in biosecurity would affect the outcome as the pigs in this farm had higher levels of exposure to both residues of dead animals and blood from subclinically infected animals (via regrouping of animals, slaughter, burial) than in most settings. The extensive environmental contamination further supports this assumption.

The farm manager’s expressed feelings of stress and depression during the outbreak could have been one of the reasons behind the habit of not immediately euthanizing or slaughtering pigs that fell ill. The farm manager stated that he did not euthanize sick pigs “because they might recover.” Healthy pigs were further put together in pens called “isolation units” and sick pigs put together in other pens. As pigs were not individually marked, this routine made the tracking of cases more difficult and, in addition, probably contributed to disease transmission. It is worth mentioning that standard disease control advice such as isolating sick pigs will be useless, or even counterproductive, if implemented when disease transmission has already started. In order for managers to make correct decisions in outbreak situations, they need knowledge about disease transmission and infection. The case fatality rate after infection with highly virulent ASFV, such as the genotype IX circulating in Uganda, is very high (Plowright et al. 1994; Gallardo et al. 2011; Chenais et al. 2015b), and the highest viral loads are observed when pigs express clinical signs (Davies et al. 2015). Thus, early euthanasia or safe slaughter must be considered key for reducing disease transmission and the total amount of virus in circulation. Given the local situation with widespread poverty and related protein deficiency, and considering that ASF is not a zoonotic disease, the authors argue that legal, safe emergency slaughter of selected animals would be a better alternative (Thomson et al. 2004; Naziri et al. 2015) than the current common practice of panic sales and illegal slaughter (Chenais et al. 2015a; Leslie et al. 2015; Nantima et al. 2016). In addition, the infectiousness of ASF (measured as *R*_0_) is not extremely high; Guinat et al. (2014) describes low to moderate transmissibility between pigs. This means that transmission can be interrupted if strict biosecurity, including the immediate removal of all infectious pigs, is exercised.

The continuous circulation and frequent outbreaks of ASF in the smallholder pig sector in Uganda impact negatively on the livelihoods of poor smallholders (Fasina et al. 2012; Chenais et al. 2015a). However, the results of this study showed that such impacts are not restricted to smallholders only. The study farm did not only suffer substantial economic loss but aspirations and non-financial investments were also shattered. As the study farm was meant to serve as a teaching farm, the failure regarding the loss of the stock and to control the disease could also have a larger impact on the society. This was further accentuated as the study farm was meant to finance the humanitarian activities of the NGO.

In order to achieve ASF control globally, nationally, and locally, many different aspects of the epidemiology need to be considered (Costard et al. 2009b). One important piece of the puzzle is biosecurity advice that is successfully implemented in the local context (Coffin et al. 2015). The authors consider simplicity, adaptability, acceptance, and cost-effectiveness to be vital for success in this regard. One example is the practice of slaughter and sale of sick pigs, as mentioned above (Chenais et al. 2015a; Leslie et al. 2015; Nantima et al. 2016). As these practices are common, they need to be considered while formulating disease control information. The fact that this practices violates national animal health laws and regulations should not automatically cause this line of enquiry to be dismissed (Leach and Scoones 2013; Coffin et al. 2015). If such regulations are unenforceable and counterproductive, then changes in policy should be investigated. Another example from the present study of adoption of biosecurity advice to the local context is the simple recommendation to implement biosecurity barriers with the associated change of boots instead of disinfectant boot baths. The larger context in which the pig farms exist cannot be neglected. Poverty will have a strong impact on the feasibility of biosecurity routines, the education level of employees, as well as the employer’s ability to maintain working conditions that favor stability and compliance with routines. Practical biosecurity routines that are attainable is necessary; the endemic status means that theoretical policies that are not implementable will quickly be revealed by continuous outbreaks.

In conclusion, at first, the study farm appeared to have the biosecurity measures in place to prevent an ASF outbreak on the farm, despite being located in an endemic region. However, the study shows that in-depth, practical knowledge in many areas is required in order to succeed with pig farming in this setting without introducing ASF. More specifically, a high level of awareness about the surrounding circumstances and the required biosecurity is fundamental. In addition, emphasis must be put on effective, feasible, and vigilant maintenance of biosecurity routines.

## References

[CR1] Amass, S., and Ragland, D., 2001. Evaluation of the efficacy of a peroxygen compound, Virkon(R)S, as a boot bath disinfectant. Journal of Swine Health and Production, 121–123

[CR2] Amass S (2000). Evaluating the efficacy of boot baths in biosecurity protocols. Journal of Swine Health and Production.

[CR3] Atuhaire DK (2013). Molecular characterization and phylogenetic study of African swine fever virus isolates from recent outbreaks in Uganda (2010–2013). Virology journal.

[CR4] Barongo, M.B. et al., 2015. Estimating the basic reproductive number (R0) for African swine fever virus (ASFV) transmission between pig herds in Uganda, PloS one, 10, e0125842 10.1371/journal.pone.0125842 PMC4418717 25938429

[CR5] Blome S, Gabriel C, Beer M (2013). Pathogenesis of African swine fever in domestic pigs and European wild boar. Virus research.

[CR6] Chenais, E. et al., 2015a. Knowledge, attitudes and practices related to African swine fever within smallholder pig production in Northern Uganda. Transboundary and emerging diseases, tbed.12347 10.1111/tbed.12347 25876769

[CR7] Chenais, E. et al., 2015b. African swine fever in Uganda: qualitative evaluation of three surveillance methods with implications for other resource-poor settings. Frontiers in Veterinary Science, 2 10.3389/fvets.2015.00051 PMC4673915 26664978

[CR8] Coffin JL (2015). A One Health, participatory epidemiology assessment of anthrax (*Bacillus anthracis*) management in Western Uganda. Soc Sci Med.

[CR9] Costard S (2013). Epidemiology of African swine fever virus. Virus research.

[CR10] Costard, S. et al., 2009a. Multivariate analysis of management and biosecurity practices in smallholder pig farms in Madagascar. Preventive veterinary medicine, 92, 199–209 10.1016/j.prevetmed.2009.08.010 PMC2806948 19781801

[CR11] Costard, S. et al., 2009b. African swine fever: how can global spread be prevented? Philosophical transactions of the Royal Society of London. Series B, Biological sciences, 364, 2683–2696 10.1098/rstb.2009.0098 PMC2865084 19687038

[CR12] Davies, K. et al., 2015. Survival of African swine fever virus in excretions from pigs experimentally infected with the Georgia 2007/1 isolate. Transboundary and emerging diseases, tbed.12381 10.1111/tbed.12381 PMC5347838 26104842

[CR13] de Balogh K, Halliday J, Lubroth J (2013). Integrating the surveillance of animal health, foodborne pathogens and foodborne diseases in developing and in-transition countries. Revue scientifique et technique.

[CR14] Dione, M. et al., 2014. Participatory assessment of animal health and husbandrypractices in smallholder pig production systems in three highpoverty districts in Uganda, Preventive veterinary medicine, 117, 565–576 10.1016/j.prevetmed.2014.10.012 25458705

[CR15] Dione, M.M. et al., 2015. Risk factors for African swine fever in smallholder pig production systems in Uganda. Transboundary and emerging diseases, tbed.12452 10.1111/tbed.12452 26662861

[CR16] FAO. The Progressive Control Pathway for FMD control (PCP-FMD) Principles, Stage Descriptions and Standards. 2014. http://www.fao.org/ag/againfo/commissions/eufmd/commissions/eufmd-home/progressive-control-pathway-pcp/en/. Accessed 20 Aug 2015

[CR17] FAOSTAT. 2013. http://faostat3.fao.org/faostat-gateway/go/to/home/E. Accessed 21 Oct 2014

[CR18] Fasina FO (2012). Cost implications of African swine fever in smallholder farrow-to-finish units: economic benefits of disease prevention through biosecurity. Transboundary and emerging diseases.

[CR19] Fernandez-Pinero J (2013). Molecular diagnosis of African swine fever by a new real-time PCR using universal probe library. Transboundary and emerging diseases.

[CR20] Gallardo C (2011). Genotyping of African swine fever virus (ASFV) isolates associated with disease outbreaks in Uganda in 2007. African Journal of biotechnology.

[CR21] Gallardo, C. et al., 2015. Experimental infection of domestic pigs with African swine fever virus Lithuania 2014 genotype II field isolate. Transboundary and emerging diseases, tbed.12346 10.1111/tbed.12346 25808027

[CR22] Greig A, Plowright W (1970). The excretion of two virulent strains of African swine fever virus by domestic pigs. J Hyg (Lond).

[CR23] Guinat C (2014). Dynamics of African swine fever virus shedding and excretion in domestic pigs infected by intramuscular inoculation and contact transmission. Veterinary research.

[CR24] Halasa, T. et al., 2016. Simulation of Spread of African Swine Fever, Including the Effects of Residues from Dead Animals, Front Vet Sci, 3, 6 10.3389/fvets.2016.00006 PMC4735426 26870740

[CR25] Ikwap, K. et al., 2014. Characterization of pig production in Gulu and Soroti districts in northern and eastern Uganda. Livestock Research for Rural Development, 26, Article #74

[CR26] Jacobsen, K. et al., 2006. Using microenterprise interventions to support the livelihoods of forcibly displaced people: the impact of a microcredit program in DP camps in Lira, Northern Uganda, Refugee Survey Quarterly, 25, 23–39

[CR27] Jori F (2013). Review of the sylvatic cycle of African swine fever in sub-Saharan Africa and the Indian ocean. Virus research.

[CR28] Leach M, Scoones I (2013). The social and political lives of zoonotic disease models: narratives, science and policy. Soc Sci Med.

[CR29] Leslie EE (2015). A description of smallholder pig production systems in eastern Indonesia. Preventive veterinary medicine.

[CR30] Montgomery E (1921). On a form of swine fever occurring in British East Africa (Kenya colony). Journal of comparative pathology and therapeutics.

[CR31] Muhangi D (2015). A longitudinal survey of African swine fever in Uganda reveals high apparent disease incidence rates in domestic pigs, but absence of detectable persistent virus infections in blood and serum. BMC Veterinary Research.

[CR32] Mur, L. et al., 2014. Thirty-five-year presence of African swine fever in Sardinia: history, evolution and risk factors for disease maintenance. Transboundary and emerging diseases, tbed.12264 10.1111/tbed.12264 25212957

[CR33] Muwonge A (2012). African swine fever among slaughter pigs in Mubende district, Uganda. Tropical animal health and production.

[CR34] Nantima N (2016). Enhancing knowledge and awareness of biosecurity practices for control of African swine fever among smallholder pig farmers in four districts along the Kenya-Uganda border. Tropical animal health and production.

[CR35] Nantima N (2015). Risk factors associated with occurrence of African swine fever outbreaks in smallholder pig farms in four districts along the Uganda-Kenya border. Tropical animal health and production.

[CR36] Naziri, D., Rich, K.M., and Bennett, B., 2015. Would a commodity‐based trade approach improve market access for Africa? A case study of the potential of beef exports from communal areas of Namibia, Development Policy Review, 33, 195–219

[CR37] NEPAD and FAO. Livestock Development Project—bankable investment project profile 2004. 2004. http://www.fao.org/docrep/007/ae562e/ae562e00.htm. Accessed 1 Oct 2014

[CR38] Penrith ML, Vosloo W (2009). Review of African swine fever: transmission, spread and control. Journal of the South African Veterinary Association.

[CR39] Penrith ML (2013). African swine fever virus eradication in Africa. Virus research.

[CR40] Plowright W, Thomson GR, Neser JA, Coetzer JAW, Thomson GR, Tustin RC (1994). African swine fever. Infectious diseases in livestock with special reference to Southern Africa, 1994.

[CR41] Sanchez-Vizcaino JM, Mur L, Martinez-Lopez B (2013). African swine fever (ASF): five years around Europe. Veterinary microbiology.

[CR42] Skaarup, T., 1985. Slaughterhouse cleaning and sanitation. In: A.a.C. Protection (ed), FAO Animal Production and Health 1985. Food and Agriculture Organization of the United Nations, Rome

[CR43] Thomson GR (2004). International trade in livestock and livestock products: the need for a commodity-based approach. Vet Rec.

[CR44] Turner C, Williams SM, Wilkinson PJ (1999). Recovery and assay of African swine fever and swine vesicular disease viruses from pig slurry. J Appl Microbiol.

[CR45] UBOS. The national livestock census report 2008. 2008. http://www.agriculture.go.ug/userfiles/National%20Livestock%20Census%20Report%202009.pdf. Accessed 22 Sep 2016

[CR46] UBOS. National population and housing census 2014. Provisional results. 2014. 2014. http://unstats.un.org/unsd/demographic/sources/census/2010_PHC/Uganda/UGA-2014-11.pdf. accessed 19 March 2015

[CR47] Young, J.R. et al., 2013. Improving smallholder farmer biosecurity in the Mekong Region through change management. Transboundary and emerging diseases, doi:10/1111/tbed.12181 10.1111/tbed.12181 26302253

